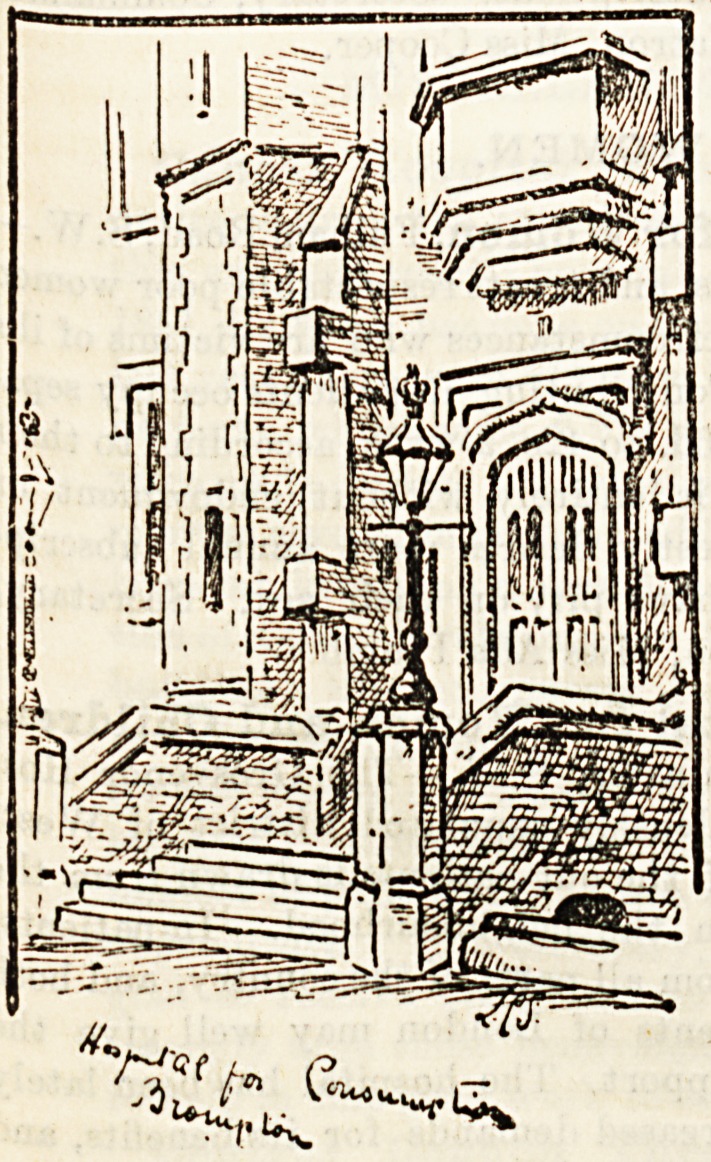# Consumption

**Published:** 1893-12-23

**Authors:** 


### SPECIAL HOSPITALS.

### CONSUMPTION.

**Brompton Hospital for Consumption and Diseases of the Chest**, S.W. — This hospital is well known as one where the patients are well cared for, and every effort is made to brighten their clouded lives. The maintenance of the new extension building entails an extra expenditure of several thousand pounds a year, and funds are urgently needed to meet this outlay. Many large annual subscriptions having been lost during the past year through the decease of old supporters, the committee are most anxious that their places should be filled up by other kind friends. Secretary, Mr. W. H. Theobald.

**City of London Hospital for Diseases of the Chest**, Victoria Park, E.—The average expenditure is about £10,000, towards which only £370 of the income is assured. Altogether about 1,000 in-patients and 15,000 out-patients are treated yearly. Assistance is much required. While the ordinary expenditure has exceeded the income for the year 1893, large demands have been made on the resources of the charity to meet exceptional expenditure for necessary sanitary improvements which have been recently carried out. Secretary, Mr. T. Storrar-Smith; Matron, Miss H. G. Hetherington.

**North London Hospital for Consumption**, Mount Vernon, Hampstead, N.W.—This institution was thoroughly reorganised and remodelled some years ago, and has now become one of the most useful of its class. Though not so large as the above, yet 400 in, and 3,000 out patients are admitted each year. Secretary, Mr. Lionel F. Hill, M.A.; Matron, Miss K. Elphick.

**Royal Hospital for Diseases of the Chest**, City Road, E.C.—This is the oldest consumption hospital in Europe, having been founded by Her Majesty's father, the late Duke of Kent, in 1814. There are 80 beds, and last year 542 in-patients and 9,324 out-patients were treated. The expenditure exceeds £6,000, towards which there is an annual subscription list of £2,000, and dividends amounting to about £100. Two additional wards have recently been opened after having been closed for seven years, and funds are urgently needed. Ten guineas constitutes life governorship, and annual subscribers of three guineas and upwards receive both in and out-patient letters. Donations will be gratefully acknowledged by the Secretary, or may be sent direct to the Chairman of the hospital, Mr. T. Andros de la Rue.

## Figures and Tables

**Figure f1:**